# Human Leukocyte Antigen (HLA) Haplotype Does Not Influence the Inflammatory Pattern of Duodenal Lymphocytosis Linked to Irritable Bowel Syndrome

**DOI:** 10.3390/medicina56120660

**Published:** 2020-11-29

**Authors:** Giuseppe Losurdo, Alessia Todeschini, Floriana Giorgio, Domenico Piscitelli, Antonio Giangaspero, Enzo Ierardi, Alfredo Di Leo

**Affiliations:** 1 Section of Gastroenterology, Department of Emergency and Organ Transplantation, University “Aldo Moro” of Bari, 70124 Bari, Italy; ale_tode@hotmail.com (A.T.); flomic@libero.it (F.G.); antoniogiangaspero@libero.it (A.G.); ierardi.enzo@gmail.com (E.I.); alfredo.dileo@uniba.it (A.D.L.); 2 PhD Course in Organs and Tissues Transplantation and Cellular Therapies, Department of Emergency and Organ Transplantation, University “Aldo Moro” of Bari, 70124 Bari, Italy; 3 Section of Pathology, Department of Emergency and Organ Transplantation, University “Aldo Moro” of Bari, 70124 Bari, Italy; domenico.piscitelli@uniba.it

**Keywords:** irritable bowel syndrome, inflammation, duodenal lymphocytosis, intraepithelial lymphocytes, smoking

## Abstract

*Background and objectives:* Duodenal lymphocytosis (DL) is a condition characterized by enhanced infiltration of intraepithelial lymphocytes (IELs) in the duodenal mucosa, and it can be linked to both gluten- and non-gluten-related diseases, such as irritable bowel syndrome (IBS). *Materials and methods:* We retrospectively selected patients with DL linked to IBS. Formalin-embedded biopsy samples of the duodenum were collected. CD3 lymphocyte immunohistochemistry was used for IELs. The real-time polymerase chain reaction was used to quantify the amount of mRNA coding for tissue transglutaminase 2 (tTG2), interferon-gamma (IFNγ), toll-like receptor 2 (TLR2), and myeloid differentiation primary response 88 (MyD88). All subjects underwent DQ2-8 haplotype analysis. Controls were represented by subjects with IBS without DL. *Results:* Thirty-two patients with IBS-DL were retrospectively recruited. Fourteen subjects (43.8%) had a DQ2-8 haplotype. DQ2-8 positive subjects had similar levels compared to negative ones for tTG2, IFNγ, TLR2, and MyD88. Cigarette smoke did not influence molecular expression in our study. Smokers had a statistically higher IELs count than non-smokers (54.2 ± 7.7 vs. 36.0 ± 8.8, *p* < 0.001). A significant, direct correlation between IELs and duodenal expression of IFNγ was found (r = 0.36, *p* = 0.04). *Conclusions:* IBS with DL showed higher expression of inflammatory markers than controls, but DQ2-8 haplotype did not seem to affect their expression. Smoking might increase IELs infiltration.

## 1. Introduction

The introduction should briefly place the study in a broad context and highlight why is duodenal lymphocytosis (DL) is a condition hallmarked by an abnormal increase of intraepithelial lymphocytes (IELs) in the duodenal mucosa, with an infiltrate of more than 25 IELs/100 enterocytes [1]. Recently, a consensus conference suggested using the term microscopic enteritis (ME) to describe this condition [2]. It may underlie either celiac disease and other gluten and non-gluten-dependent disorders, such as food allergy, inflammatory bowel disease, immunoglobulin deficiencies, drugs, *Helicobacter pylori*, and other infective gastrointestinal conditions, as well as irritable bowel syndrome (IBS) [3].

IBS is a chronic gastrointestinal functional syndrome characterized by abdominal pain and alteration of the frequency and form of stools [4]. In particular, according to the Rome IV foundation report [5], abdominal pain associated with defecation or a change in bowel habits should be present at least 1 day per week during the preceding month, and all symptoms should not be related to any organic disease. The hypotheses to explain the pathogenesis of IBS are very different [6]. Exposure to drugs, such as antibiotics, or to infectious gastroenteritis may trigger its onset [6]. A neural dysfunction with increased sensitivity to colon distension has been hypothesized [6]. Psychosocial factors, such as the co-existence of anxiety and depression, personality abnormalities, and stress, are known to aggravate symptoms [6]. Some genetic polymorphisms in ion transporters have been associated with a high risk of IBS [6]. A perturbation of gut microbiota is another possible predisposing factor [6]. Finally, the hypothesis that a local inflammation could underlie IBS is an intriguing suggestion. In this regard, several studies have shown that a local inflammatory state can be the basis of this disease [7]. Barbara et al. in this regard, demonstrated a crowding of mast cells in degranulation near the nerves of the mucosa [8].

On these bases, many studies have shown infiltration of IELs in the epithelium of the duodenum of patients with IBS. Spiller et al. demonstrated for the first time that an increase in IELs compared to controls occurred in post-infectious diarrheal IBS, simultaneously with an increase in intestinal permeability [9]. Sundin et al. [10] found high levels of aberrant mucosal CD4+/CD8+ lymphocytes in the lamina propria and colon mucosa in patients with post-infectious IBS. In a series of 100 DL cases, Aziz et al. [11] reported a prevalence of irritable bowel syndrome of 18%. Remes Troche et al. [12] discovered an average number of IELs in IBS of 16.7 ± 6 per 100 enterocytes, much lower than that observed in celiac disease. An over-expression of duodenal IELs has also been shown in the constipation subclass of IBS [13], thus inducing to speculate that a microscopic inflammatory alteration could trigger this “functional” disorder. However, the abnormal infiltration of IELs in IBS patients may induce a misdiagnosis of seronegative celiac disease, based on the histological picture. Indeed, an overdiagnosis of celiac disease is frequent when relying only on histology or serology without considering specific guidelines [14]. This aspect is relevant since immunity patterns of IBS and celiac disease are almost different. Among the molecules that play a role in the immune response induced by the gluten, tissue transglutaminase 2 (tTG2), interferon-gamma (IFNγ), toll-like receptor 2 (TLR2), and myeloid differentiation primary response 88 (MyD88) should be mentioned. In a previous experience [15], we showed that mucosal high levels of tTG2 and IFNγ mRNA might forecast the onset of celiac disease more than gluten sensitivity with high specificity, while MyD88 levels could suggest that intestinal permeability was more increased when severe intestinal damage underlay DL in both gluten-related and unrelated conditions.

Herein, we aimed to assess the expression of inflammatory markers linked to the celiac disease on biopsy samples of the duodenum in a cohort of patients affected by DL linked to IBS.

## 2. Materials and Methods

### 2.1. Patients

We retrospectively enrolled patients, from March to September 2013, affected by DL of different causes defined according to the criteria of the Bucharest Consensus Conference [2]. We excluded subjects with celiac disease based on medical history along with the positivity of IgA anti-tissue transglutaminase 2 (anti-tTG2) or anti-endomysium antibodies (Bio-Rad Laboratories, Inc., Segrate, MI, Italy). The subjects with wheat allergy (diagnosed by skin prick test and IgE anti-gliadin, (Bio-Rad Laboratories, Inc., Segrate, MI, Italy)) and subjects with a diagnosis of non-celiac gluten sensitivity (self-reported or according to the Salerno criteria [16]) were also excluded. Finally, we excluded patients with DL due to non-gluten-related organic causes. In detail, we excluded subjects with a medical history of vasculitis, inflammatory bowel disease, active *H. pylori*, or other gastrointestinal infections. We excluded subjects with immunoglobulin deficiencies and self-reported use of drugs, such as non-steroidal anti-inflammatory drugs or olmesartan. Furthermore, all patients underwent glucose breath test to rule out small intestinal bacterial overgrowth. Overall diagnostic work-up of DL has been described in a previous report [17]. In detail, at baseline, all patients underwent serology for CD (IgG and IgA tTG2, anti-endomysium antibody EMA) (Bio-Rad Laboratories, Inc., Segrate, MI, Italia). We obtained blood samples for full blood count, folate, vitamin B12, serum protein electrophoresis with genotyping human leukocyte antigens (HLA). The follow-up strategy reckoned on some investigations, such as stool investigations, to detect bowel infection or parasitic infestation (particularly Giardia Lambia), fecal occult blood test (Hemoccult, Beckman Coulter, Cassina de ‘Pecchi, MI, Italia), calprotectin (CAL Detect^®^, Sofar SpA, Trezzano Rosa, MI, Italia). If a fecal occult blood test or calprotectin were positive, colonoscopy with random biopsy samples and/or enteric MRI were performed. We used lactose and glucose breath test to rule out small intestinal bacterial overgrowth (SIBO) and lactose malabsorption, respectively. After applying all inclusion and exclusion criteria, we enrolled patients with IBS, fulfilling Rome IV criteria [5]. The control group was represented by patients with IBS without DL features on duodenal histological examination.

The study was performed in agreement with the Declaration of Helsinki. Being a retrospective study, which did not need investigations other than what was required for the clinical management and diagnosis of IBS, the study was reviewed and approved after a meeting of the authors, all affiliated with the Gastroenterology Unit of Bari University Hospital Policlinico (Italy). Written informed consent to undergo an esophagogastroduodenoscopy (in the Italian language) was obtained from each patient. In the text, it was clearly reported: “I accept that demographic and clinical data and the outcome of the procedure can be managed anonymously for scientific purposes”.

### 2.2. Histology and Immunohistochemistry

At baseline, the histological analysis had been performed on hematoxylin-eosin stained sections. Immunohistochemistry of CD3 (Novocastra Leica Biosystems, Newcastle Ltd., Newcastle, UK) lymphocytes was performed to depict IELs.

### 2.3. Molecular Analysis

Real-time polymerase chain reaction (RT-PCR) was used to detect the amount of mRNA coding for tTG2, IFNγ, TLR2, and MyD88 in duodenal tissue samples, as previously described [18].

Paraffin block of biopsy samples was cut in 10 sections of 10 μm, and RNA was extracted using the RNeasy FFPE Kit (Qiagen, GmbH, Hilden, Germany) according to the manufacturer’s instruction. Finally, the mRNA concentrations were estimated by spectrophotometric examination at 260 nm. Aliquots of total mRNA (1 mg) were reverse-transcribed using random hexamers (AppliedBiosystems, Monza, Italia) with 3125 U/dL of reverse transcriptase (MultiScribe, AppliedBiosystems, Monza, Italia) in a final volume of 50 μL.

RT-PCR, a quantitative methodic that allows studying gene expression, was performed on plates with 96 wells on Detection System ABI Prism 7900HT Sequence (Applied Biosystems, Monza, Italia). The data collection and analysis were performed using the machine’s software. At first, the RT-PCR amplification was performed with a cDNA strand with a final concentration of 1× TaqMan primers and 1× di TaqMan Universal PCR Master Mix in a final reaction volume of 50 μL. Every sample was analyzed in triplicate (all experiments were repeated twice). A non-template control (RNase-free water) was included on every plate. Specific thermal cycler conditions were 2 min at 50 °C (UNG activation), 10 min at 95 °C, followed by 40 cycles of 15 s at 95 °C and 1 min at 60 °C. A standard curve plus validation experiment was performed for each primer/probe set (Table 1). A series of dilutions (from 20 to 0.1 ng/μL) of cDNA was used as a stamp for each primer/probe set. Standard curves were generated with a threshold cycle (CT) of numerical values against cDNA log quantity. Then, the average amount of target genes was normalized to a reference gene (GAPDH) and a calibrator. Finally, for each sample, relative quantization was drawn by a bar chart using Microsoft Excel. The assays were provided as a mixture of 20× PCR primers and TaqMan Minor Groove Binder 6-FAM probes marked at 3′ end with a non-fluorescent quencher colorant.

Considering the average values of the expression of these two molecules in negative controls as 1, mRNA levels were expressed as fold-change. Then, the expression of these markers was related to genetic factors (HLA haplotype), environmental factors (cigarette smoking), and clinical factors (symptoms and vitamin deficiencies).

### 2.4. Statistical Analysis

All variables were tested for normality by the Kolmogorov-Smirnov test, which confirmed Gaussian distribution. Comparison between continuous data was performed by Student’s *t*-test, and comparison among discrete variables by Fisher’s exact test, χ^2^ test with Yates correction. The correlation between continuous data was assessed by Pearson’s test. Values of *p* < 0.05 were considered significant. Statistical analyses were performed using the statistical software SPSS version 21 and GraphPad Prism version 5.00 for Windows.

## 3. Results

### 3.1. Patients Baseline Features

**Figure 1:**
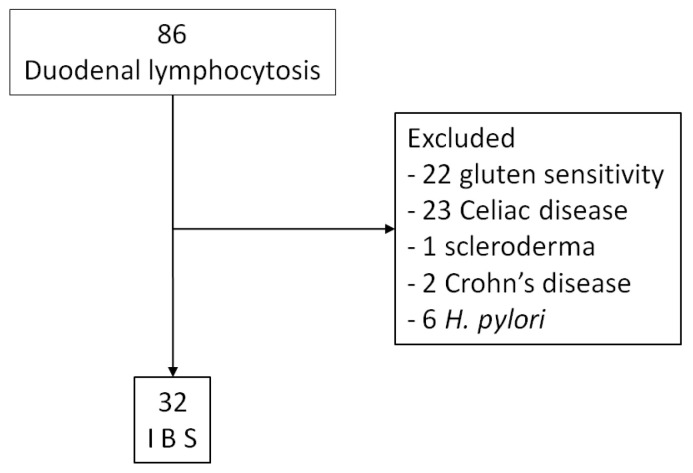
Flowchart of patient enrollment. IBS: irritable bowel syndrome.

Among a group of 86 consecutively enrolled patients with different causes of DL, we selected 32 subjects with IBS. Details are shown in the flowchart in Figure 1. The prevalence of IBS among all DL was 37.2%. 

Enrolled patients were mainly female (75%), and they had a mean age of 36.4 ± 14.1 years (range 18–71). Five of them were smokers (15.6%). Only one patient had a first-degree relative with celiac disease, even if 14 (43.8%) were carriers of DQ2/8 haplotype. Three patients had anti-nuclear antibody (ANA) low-titer positivity (1:80); two had IgA anti-gliadin (AGA) positivity; six (18.8%) were affected by Hashimoto’s thyroiditis; no other autoimmune disease was reported. Blood tests demonstrated anemia in 18.8% of patients, and folate and B12 deficiency in 28.1% and 6.3%, respectively; none had atrophic gastritis. In six patients with anemia, a colonoscopy showed a normal picture, except for one case in which two diminutive polyps were removed. The most commonly reported symptoms were abdominal pain (referred in all patients as diagnostic criteria for IBS), weight loss, bloating, diarrhea, fatigue, or anxiety.

Two patients displayed an increased value of C-reactive protein (CRP) associated with neutrophilic leukocytosis, not explainable by any infectious cause, despite a spontaneous normalization was observed after one month. In all patients, histological analysis showed at least 25 IELs/100 enterocytes (range 25–65), on average 38.8 ± 10.8. These details are reported in Table 2. 

In the control group, 15 patients affected by IBS with duodenal biopsy specimens with IELs count lower than a threshold of 25/100 enterocytes were enrolled. This control group had clinical features similar to the study group, except for IELs count, as shown in Table 3.

### 3.2. Expression of Molecules in Patients with DL Due to IBS

RT-PCR showed that all studied molecules were overexpressed in the duodenal mucosa of patients with IBS and DL than IBS alone. In Table 4, all molecular values are expressed as fold-change compared to controls. 

### 3.3. Influence of DQ2/8 Haplotype on Molecular Profile

**Figure 2:**
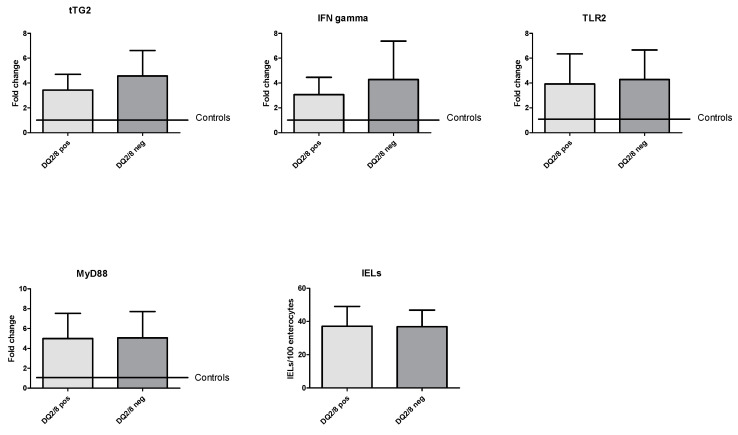
Molecular expression of coding mRNA of tTG2, IFNγ, TLR2, and MyD88 and IELs count based on HLA haplotype. HLA: human leukocyte antigen; IELs: intraepithelial lymphocytes; IFNγ: interferon-gamma; MyD88: myeloid differentiation primary response 88; TLR2: toll-like receptor 2; tTG2: tissue transglutaminase 2; pos: positive; neg: negative.

DL patients with HLA DQ2/8 (*n* = 14) expressed tTG2 levels comparable to DL DQ2/8-negative subjects (3.4 ± 1.3 vs. 4.6 ± 2.0, *p* = 0.08), but statistically higher than controls (1.1 ± 0.2, *p* < 0.001). Similarly, the levels of IFNγ (4.3 ± 3.1 vs. 3.4 ± 1.3, *p* = 0.19), TLR2 (4.3 ± 2.4 vs. 3.9 ± 2.4, *p* = 0.69), and MyD88 (5.1 ± 2.6 vs. 5.0 ± 2.5, *p* = 0.94) in patients with DL DQ2/8 were similar to those in DL DQ2/8-negative patients. In any case, levels of the above-mentioned molecules were higher in IBS-DL than IBS without DL controls. Furthermore, we did not find any correlation for IELs count based on DQ2/8 haplotype status (38.7 ± 10.4 vs. 38.9 ± 11.7, *p* = 0.97). In Figure 2, histograms summarize these analyses.

### 3.4. Influence of Smoking on Molecular Profile

**Figure 3:**
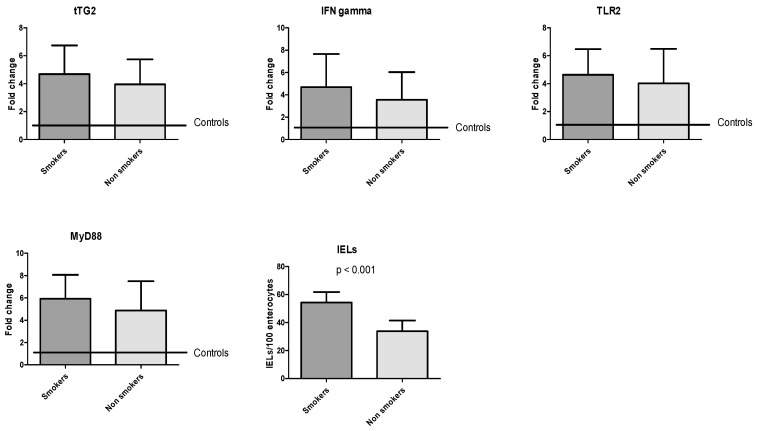
Molecular expression of coding mRNA of tTG2, IFNγ, TLR2, and MyD88 and IELs count based on smoker or non-smoker status.

Cigarette smoke did not influence molecular expression in our study. Smokers and non-smokers expressed same levels of mRNA for tTG2 (4.7 ± 2.5 vs. 3.9 ± 1.8, *p* = 0.49), IFNγ (4.7 ± 2.9 vs. 3.9 ± 1.8, *p* = 0.46), TLR2 (4.6 ± 1.8 vs. 4.0 ± 2.5, *p* = 0.54), and MyD88 (5.9 ± 2.1 vs. 4.9 ± 2.6, *p* = 0.36). Instead, smokers had an IELs count statistically higher than non-smokers (54.2 ± 7.7 vs. 36.0 ± 8.8, *p* < 0.001), as seen in Figure 3.

### 3.5. Influence of Hashimoto’s Thyroiditis on Molecular Profile

Six patients were affected by Hashimoto’s thyroiditis. On that note, subjects with Hashimoto’s thyroiditis did not have higher IELs count than subjects without thyroiditis (39.3 ± 6.8 vs. 38.7 ± 11.7, *p* = 0.90). Finally, patients with thyroiditis had similar levels of mRNA encoding for studied molecules as patients without thyroiditis for tTG2 (3.9 ± 1.6 vs. 3.9 ± 1.8, *p* = 0.87), IFNγ (3.2 ± 1.4 vs. 3.8 ± 1.7, *p* = 0.43), TLR2 (4.8 ± 3.1 vs. 3.9 ± 2.2, *p* = 0.56), and MyD88 (5.4 ± 2.7 vs. 4.9 ± 2.6, *p* = 0.69). 

### 3.6. Influence of Other Clinical Factors on Molecular Profile

Other clinical and pathologic features (anemia, folate or B12 deficiency, diarrhea, bloating, etc.) did not show any significant correlation between these variables and the expression of mRNA or IELs count. Patients with fatigue displayed slightly higher IELs count (50.7 ± 18.3 vs. 37.6 ± 9.4, *p* = 0.045). 

### 3.7. Correlation between IELs Count and Molecular Expression

**Figure 4:**
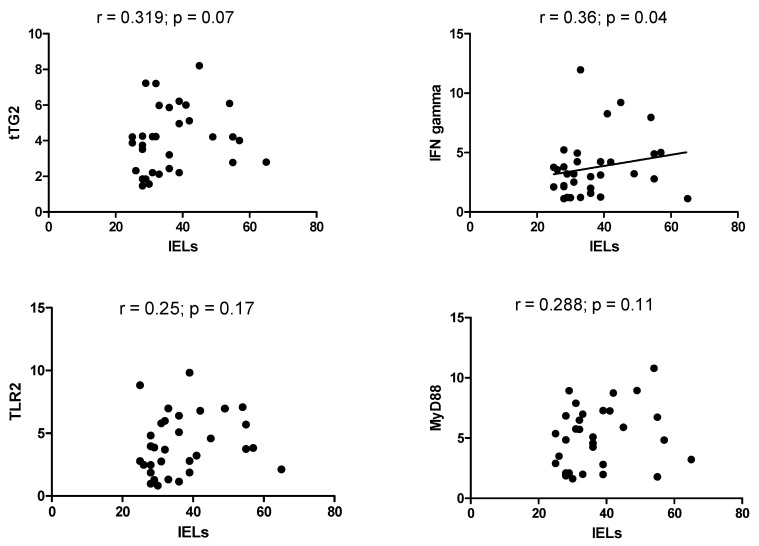
Scatterplot between IELs density and mRNA molecular expression of tTG2, IFNγ, TLR2, and MyD88.

Scatterplots, reported in Figure 4, revealed a significant correlation between IELs count and duodenal expression of IFNγ, with r = 0.36 (*p* = 0.04). On the other hand, there was no significant correlation with other markers, in particular between IELs and tTG2 (r = 0.319, *p* = 0.07), TLR2 (r = 0.25, *p* = 0.17) and MyD88 (r = 0.288, *p* = 0.11). 

## 4. Discussion

IBS is the most common so-called “functional” gastrointestinal disorder. In Italy, its estimated prevalence is about 3.5%, according to the Italian society of general medicine (SIMG) [19]. Although IBS remains a functional condition, its heterogeneous pathogenesis seems to imply alterations in motility, visceral nociceptive sensitivity, microbiome, dietary factors, infectious factors, and low-grade mucosal inflammation [20]. Moreover, immune activation may presumably be involved in patients with functional gastrointestinal disorders.

In functional dyspepsia, several studies have underlined increased eosinophils and IELs count in duodenal mucosa [13,21,22], suggesting these abnormalities as a possible primum movens in its pathogenesis [23]. However, few studies have investigated this issue in IBS [24]. Arevalo et al. demonstrated an increased prevalence of intestinal lymphocytosis compared to controls [25]. In four further studies [26,27,28,29], only two demonstrated an increase of IELs compared to healthy controls [26,27]. Although Martinez et al. did not demonstrate a higher lymphocytosis rate in the jejunal mucosa of patients with IBS, they found an increase of mast cells and a decreased mRNA codifying for some tight junction components (particularly ZO1 and ZO3), which are markers of impaired intestinal permeability [30]. On the other side, different studies evaluated pro-inflammatory cytokine expression in colonic mucosa and inflammatory infiltrate. Barbara et al. [8,31] showed increased infiltration of degranulating mast cells in the colonic mucosa, which was more intense close to sensitive nerve terminals. Furthermore, Sundin [10] showed a shift of CD3+ lymphocytes to CD4+CD8+ lymphocytes. Other authors found an increased expression of inteleukin (IL)-8 in colonic mucosa [32]. Moreover, a Chinese study demonstrated an over-expression of IFNγ in the colonic mucosa in patients with post-infectious IBS [33]. Conversely, several studies reported a reduced expression of IL-10 (an anti-inflammatory cytokine) in IBS [34,35]. Based on these findings, in this study, we tested mRNA expression encoding for four molecules in paraffin-embedded duodenal tissue in patients with DL linked to IBS. These molecules (tTG2, IFNγ, TLR2, and MyD88) are directly correlated with local inflammation and are markers of tissue damage. Some of these molecules are also involved in the pathogenesis of CD and gluten-related disorders. tTG2 enzyme has a central role in CD pathogenesis. Its primary function is to catalyze the reaction of transamidation, which generates intrachain bonds between glutamine residues. In predisposed subjects, tTG2 catalyzes the deamidation of glutamine residues to glutamic acid, thus making these peptides more immunogenic and amplifying autoimmune phenomena [36]. 

Myeloid differentiation primary response 88 (MyD88) is an intracellular adapter protein that receives signals by all toll-like receptors (TLRs) and is involved in the immune response to bacterial infection [37]. However, it seems to play a role in regulating the intestinal permeability in CD. The role played by TLRs in gluten-related inflammation is confirmed by increased mRNA levels of TLR2 and TLR4 in duodenal mucosal of CD patients [38]. Gluten-mediated inflammation generates a cytokine substratum in the intestinal mucosa dominated by tumor necrosis factor-alpha (TNFα) and IFNγ [39]. IFNγ is not only a mediator of inflammation, but it increases intestinal permeability and transepithelial flow of gliadin [40]. The first relevant result in our analysis is that the four molecules we took into account were all overexpressed in patients with DL due to IBS compared to controls. These data underlined how this subgroup of patients was characterized by the presence of mild mucosal inflammation. However, as it is shown in Figure 4, only IFNγ expression correlated with IELs count. This represents a remarkable result because IFNγ is the most important inflammatory marker, while the other three molecules are mainly involved in the gliadin-mediated inflammatory response. This would testify that there is a “typical” inflammation in a subgroup of patients with IBS, unrelated to gliadin stimulation. Therefore, we may hypothesize that, despite duodenal involvement, IBS is not characterized by an immunity profile typical of celiac disease. A confirmation of this assumption could be suggested by the finding that patients with DQ2/8 haplotype did not express these molecules most intensively as patients without the haplotype (Figure 2). Such result underlined that a patient with an at-risk haplotype and Marsh stage 1 should not be considered celiac, as stated in current guidelines, because the molecular profile is comparable to a DQ2/8-negative patient, and he does not overexpress molecules involved in CD pathogenesis, as already described in previous experiences [15,41]. Unfortunately, it is common malpractice diagnosing CD based on HLA or on histology in the absence of specific positive antibodies. In this regard, a study performed in a tertiary center showed that among 105 CD patients diagnosed in non-dedicated centers, the diagnosis of CD had been rejected in 43, and seven of them were re-classified as IBS [42]. In a 9-year retrospective Italian study of 614 patients with a diagnosis of CD, 65% of subjects were under a gluten-free diet at the time of the reclassification visit, but only in 29.5%, the diagnosis was confirmed [43]. The interest of the media—particularly the Internet—in gluten-related disease and toxic properties of gluten is continuously growing, and therefore an increasing number of people take on a gluten-free diet without medical advice [44]. This has led to an exponential rise of a gluten-free diet in IBS subjects, prescribed by alternative medicine practitioners and dieticians without specific medical knowledge or applied as self-medication by the patients themselves [45,46,47]. In this regard, our study highlighted that patients with IBS and duodenal damage, independently from HLA, did not present an inflammatory pattern related to gluten injury, and a gluten-free diet would be unjustified. The second relevant finding was that smokers had a higher IELs count than the non-smoker (Figure 3). It is plenty of data about the connection between cigarette smoke and IBS; some systematic reviews concluded that there is not a strong and certain link between these two conditions [48]. However, there are many epidemiological studies that have revealed how smoke could be a risk factor for IBS (in particular when it is associated with other functional conditions) [49,50,51], and it is known that changes in lifestyle, particularly smoking cessation, positively mirror on IBS symptoms [52]. The effect of smoke on DL is poorly investigated. On the other hand, in Crohn’s disease, it is known that smoking aggravates the inflammatory mucosal damage, reduces the response to treatment, and worsens the prognosis, increasing the risk of surgery [53]. However, only a few studies have investigated the effect of smoking on IELs. In non-celiac subjects, smoking exacerbates the severity of duodenal lesions in patients taking low-dose aspirin and proton pump inhibitors [54]. In microscopic colitis, a disease characterized by an increase of IELs in the colon, smokers have more frequently a persistent disease course [55], and smoking may intensify clinical symptoms (increased number of watery stool discharges) and reduce the probability of clinical remission [56]. These data, though indirectly, show that smoking could be an aggravating factor for DL; further studies are necessary in the future to clarify the strength of this link. However, the low number of smoking patients could be a limitation in our study to draw solid conclusions. Overall, we could only recruit 32 patients, and this is a sign that the co-occurrence of IBS and DL is hard to find, and further studies are necessary.

In conclusion, the results of this investigation seem to underline how a subgroup of patients with IBS and DL show a latent and non-specific inflammatory pattern in the duodenal mucosa that is not affected by HLA. In addition, the relationship between cigarette smoke and the density of the infiltrated IELs is an interesting aspect that would deserve further study in the future, both to confirm the result and to understand if smoking cessation can hesitate in the reduction of inflammation at the level of the duodenal mucosa.

## 5. Conclusions

In conclusion, our study underlines the existence of two types of IBS: with and without duodenal DL. In the first one, a mild mucosal inflammation is spread in the whole small bowel, while it is presumable that in the second one, inflammatory alterations are more faded and limited to less extensive bowel areas.

## Figures and Tables

**Table 1:** Primers and probes used for RT-PCR.

Molecules	Primers and Probes
**Tissue transglutaminase 2**	Primer Forward:ATAAGTTAGCGCCGCTCTCC
	Primer Reverse: CGGTGGCTCCTTCCACTG
	Probe: GCCAGCCGCCAGTG
**Interferon-gamma**	Primer Forward: CGCTTTACTTTATAGAAAACCTGGA
	Primer Reverse: TCAATGAAGAGAACTTGGTCATTC
	Probe: GCTTGAATCTAAA
**Toll-like receptor 2**	Primer Forward: CAAGATTCAAAGTATTTA
	Primer Reverse: CCAGGTG CATTTAAAGA
	Probe: TGCCCCTACTCAATCT
**MyD88**	Primer Forward: CAAGGCCTTGTCCCTGC
	Primer Reverse: TCTGCCCTGCCTCCT
	Probe: AGGCCCTGGGTGTGTGT

RT-PCR: Real-time polymerase chain reaction; MyD88: myeloid differentiation primary response 88.

**Table 2:** Main characteristics of enrolled patients with DL due to IBS.

Variable	*N* (%) or Mean ± Standard Deviation
Sex M/F	8/24
Age	36.4 ± 14.1
Smokers	5 (15.6%)
IELs count	38.8 ± 10.8
ANA	3 (9.4%)
AGA	2 (6.3%)
First-degree familiarity for celiac disease	1 (3.1%)
HLA DQ2/8	14 (43.8%)
Autoimmune thyroiditis	6 (18.8%)
Iron deficiency	6 (18.8%)
Vitamin B12 deficiency	2 (6.3%)
Folate deficiency	9 (28.1%)
Weight loss	5 (15.6%)
Bloating	15 (50%)
Diarrhea	24 (75.0%)
Weakness	3 (9.4%)
Abdominal pain	32 (100%)
Anxiety	1 (3.1%)
Neutrophilic leukocytosis	2 (6.3%)
High CRP	2 (6.3%)
Hyper-ferritinemia	0 (0%)

AGA: anti-gliadin antibodies; ANA: anti-nucleus antibodies; CRP: C-reactive protein; DL: duodenal lymphocytosis; IBS: irritable bowel syndrome; IELs: intraepithelial lymphocytes; HLA: human leukocyte antigens; M: male; F: female.

**Table 3:** Comparison of main demographic, clinical, and pathologic features between study group and controls.

Variable	IBS with DL (*n* = 32)	IBS without DL (*n* = 15)	*p*
Sex M/F	8/24	3/12	1
Age	36.4 ± 14.1	37.4 ± 15.9	0.83
Smokers	5 (15.6%)	2 (13.3%)	1
IELs count	38.8 ± 10.8	13.8 ± 8.6	<0.001
ANA	3 (9.4%)	2 (13.3%)	0.65
AGA	2 (6.3%)	1 (6.6%)	1
First-degree familiarity for celiac disease	1 (3.1%)	0 (0%)	0.51
HLA DQ2/8	14 (43.8%)	5 (33.3%)	0.54
Autoimmune thyroiditis	6 (18.8%)	2 (13.3%)	1
Iron deficiency	6 (18.8%)	3 (20%)	1
Vitamin B12 deficiency	2 (6.3%)	0 (0%)	0.83
Folate deficiency	9 (28.1%)	4 (26.6%)	1
Weight loss	5 (15.6%)	3 (20%)	0.69
Bloating	15 (50%)	8 (53.3%)	0.76
Diarrhea	24 (75.0%)	9 (60.0%)	0.32
Weakness	3 (9.4%)	2 (13.3%)	0.65
Abdominal pain	32 (100%)	15 (100%)	1
Anxiety	1 (3.1%)	1 (6.6%)	0.54
Neutrophilic leukocytosis	2 (6.3%)	0 (0%)	0.83
High CRP	2 (6.3%)	0 (0%)	0.83
Hyper-ferritinemia	0 (0%)	0 (0%)	1

AGA: anti-gliadin antibodies; ANA: anti-nucleus antibodies; CRP: C-reactive protein; DL: duodenal lymphocytosis; IBS: irritable bowel syndrome; IELs: intraepithelial lymphocytes; HLA: human leukocyte antigens.

**Table 4:** Expression of mRNA coding for tTG2, IFNγ, TLR2, and MyD88 in patients with DL due to IBS and control group.

	IBS with DL (*n* = 32)	IBS without DL (*n* = 15)	*p*
**Tissue transglutaminase 2**	4.1 ± 1.8	1.1 ± 0.2	<0.001
**Interferon gamma**	3.7 ± 2.5	1.1 ± 0.3	<0.001
**Toll-like receptor 2**	4.1 ± 2.4	1.0 ± 0.1	<0.001
**MyD88**	5.0 ± 2.5	1.2 ± 0.4	<0.001

tTG2: tissue transglutaminase 2; IFNγ: interferon-gamma; TLR2: toll-like receptor 2.
